# Development and Validation of a New Wearable Mobile Device for the Automated Detection of Resting Tremor in Parkinson’s Disease and Essential Tremor

**DOI:** 10.3390/diagnostics11020200

**Published:** 2021-01-29

**Authors:** Basilio Vescio, Rita Nisticò, Antonio Augimeri, Andrea Quattrone, Marianna Crasà, Aldo Quattrone

**Affiliations:** 1 Biotecnomed S.C.aR.L., 88100 Catanzaro, Italy; basilio.vescio@biotecnomed.it (B.V.); antonio.augimeri@biotecnomed.it (A.A.); 2 Neuroimaging Unit, Institute of Molecular Bioimaging and Physiology of the National Research Council (IBFM-CNR), 88100 Catanzaro, Italy; rita.nistico@cnr.it; 3 Institute of Neurology, Magna Græcia University, 88100 Catanzaro, Italy; an.quattrone@hotmail.it; 4 Neuroscience Research Center, Magna Græcia University, 88100 Catanzaro, Italy; marianna.crasa@gmail.com

**Keywords:** electromyography, rest tremor, Parkinson’s disease, wearable device, phase pattern

## Abstract

Involuntary tremor at rest is observed in patients with Parkinson’s disease (PD) or essential tremor (ET). Electromyography (EMG) studies have shown that phase displacement between antagonistic muscles at prevalent tremor frequency can accurately differentiate resting tremor in PD from that detected in ET. Currently, phase evaluation is qualitative in most cases. The aim of this study is to develop and validate a new mobile tool for the automated and quantitative characterization of phase displacement (resting tremor pattern) in ambulatory clinical settings. A new low-cost, wearable mobile device, called µEMG, is described, based on low-end instrumentation amplifiers and simple digital signal processing (DSP) capabilities. Measurements of resting tremor characteristics from this new device were compared with standard EMG. A good level of agreement was found in a sample of 21 subjects (14 PD patients with alternating resting tremor pattern and 7 ET patients with synchronous resting tremor pattern). Our results demonstrate that tremor analysis using µEMG is easy to perform and it can be used in routine clinical practice for the automated quantification of resting tremor patterns. Moreover, the measurement process is handy and operator-independent.

## 1. Introduction

Tremor is an involuntary, rhythmic, oscillatory movement of a body part. Rest tremor is tremor in a body part that is not voluntarily activated [1]. It should be assessed when the patient is attempting to relax and is given adequate opportunity to relax the affected body part. This may require complete support of the involved body part (e.g., the arm) against gravity. In Parkinson’s disease (PD), the amplitude of rest tremor almost always diminishes or is abolished, at least transiently, during goal-directed voluntary movements, and tremor amplitude typically increases during mental stress. In addition, rest tremor may appear or increase while walking or when performing movements of another body part [2].

Resting tremor is not pathognomonic for PD and has also been observed in other neurologic diseases, such as essential tremor with resting tremor (rET). Due to these overlapping symptoms, misdiagnosis between ET and PD tremor may occur in 20–30% of cases [3].

The diagnosis of resting tremor is mainly a clinical process where patients are interviewed and undergo clinical observation. Clinical assessments are often combined with electromyography (EMG) and accelerometers measurements to determine tremor amplitude, frequency and activation pattern.

**Figure 1:**
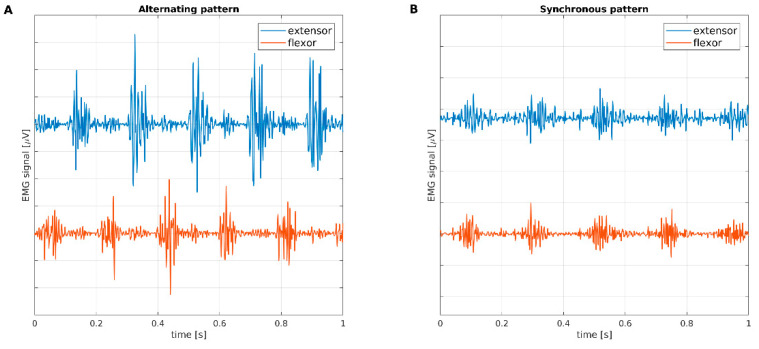
One second of EMG recording from extensor and flexor muscles of (**A**) one PD patient and (**B**) one rET patient. It can be visually assessed that bursts are phase-shifted in (**A**) and synchronous in (**B**). EMG: electromyography; PD: Parkinson’s disease; rET: essential tremor with resting tremor.

Several studies have investigated the electrophysiological parameters of resting tremor in patients with PD and ET. We previously investigated the electrophysiological characteristics of resting tremor in ET and PD patients, demonstrating that the parameter that best distinguished the two diseases was muscle activation pattern [4]. Activation pattern can be synchronous, when bursts recorded from antagonist muscles are in phase, or asynchronous, when bursts are phase-shifted, as shown in Figure 1. Synchronous activation patterns of resting tremor are typical of subjects with rET, while asynchronous patterns are observed in subjects with tPD.

Clinical evaluation of activation patterns is currently performed visually on EMG recordings. Therefore, the assessment of synchronous or asynchronous patterns is made on a qualitative basis and may lead to incorrect diagnosis, due to operator’s interpretation of phase shifting between signals.

Breit S. et al. [5] developed a long-term EMG-based automated analysis procedure for differentiating parkinsonian tremor from essential tremor in a sample of 45 subjects. They found a considerable overlap between phase data, as they considered all tremor occurrences during 24-h activity and did not focus on rest tremor only, as investigated by Nisticò et al. [4].

There is an increasing interest in methods based on accelerometers [6,7] and/or surface electromyography (EMG) electrodes, since they are readily available, non-invasive diagnostic tools.

Hossen et al. [8] introduced an approximate power spectral density, estimated by means of a wavelet decomposition with soft decision algorithm on both EMG and accelerometer signals recorded for 30 s, in order to discriminate between PD and ET patients. In this work, only postural tremor was considered and an 85% classification accuracy was achieved.

Ruonala et al. [9] tried to differentiate patients with essential tremor from patients with Parkinson’s disease using electromyographic and accelerometric data during iso-metric tension of the arms.

In other previous studies [10,11,12,13,14], mobile applications on Apple iPhone for the analysis of tremor in patients with PD and ET, performing accelerometer measurements, were tested, suggesting that those apps were a valid alternative tool to EMG in assessing the frequency of tremor. Other authors investigated the evaluation of tremor severity in PD using wearable inertial devices [15,16]; in particular, López-Blanco et al. [17] focused on rest tremor. Another work described a new device and method for the continuous and long-term monitoring of tremor due to PD. This method was based on the evaluation of frequency data from multi-axial sensors and the device appeared promising for routine clinical practice [18]. Recently, some authors [19] evaluated the presence of tremor during muscular effort and the influence of emotional stress using four tri-axial accelerometers placed on a hand’s fingers. No study, however, evaluated tremor phase pattern using wearable mobile devices in PD and ET patients with resting tremor.

In the present work, we aimed at recording resting tremor in PD and rET patients by using a miniaturized, wearable and mobile device, called µEMG (micro-EMG), capable of providing a quantitative, operator-independent and automated analysis of the tremor’s electrophysiological parameters, including tremor pattern, compared to those recorded with accelerometer and EMG surface electrodes by using classical EMG.

## 2. Materials and Methods

Fourteen patients with a diagnosis of idiopathic PD in accordance with the UK Brain Bank criteria [20] and seven patients with rET were enrolled. Only PD (tPD) and ET patients (rET) with rest tremor were enrolled in this study (Table 1). In particular, patients with Unified Parkinson Disease Rating Scale Motor Examination (UPDRS-ME) resting tremor score of ≥2 for at least one hand during the physical examination, and a history of resting tremor, were considered eligible for this study. Exclusion criteria were: (a) presence of dementia; (b) deficits in language comprehension and production; (c) use of psychoactive drugs during the last 3 months preceding the experiment; (d) major depression; (e) neurological comorbidity.

All subjects gave written informed consent before participation. All the experimental procedures were conducted according to the policies and ethical principles of the Declaration of Helsinki. The study was approved by the Ethics Committee of the Calabria Region, “Sezione Area Centro” (no. 333, 22 October 2020).

The upper limb with the dominant rest tremor was recorded. Rest measurements were performed with the patient’s arm flexed at 90°, fully supported against gravity.

Classical EMG recordings were performed using a Dantec Keypoint system, by Natus Neurology. A monoaxial accelerometer (Acceleration transducer, Natus), was placed on the dorsal side of the patient’s hand and 2 pairs of surface electrodes were positioned on the antagonistic groups of muscles of the forearm, as described elsewhere [4].

**Figure 2:**
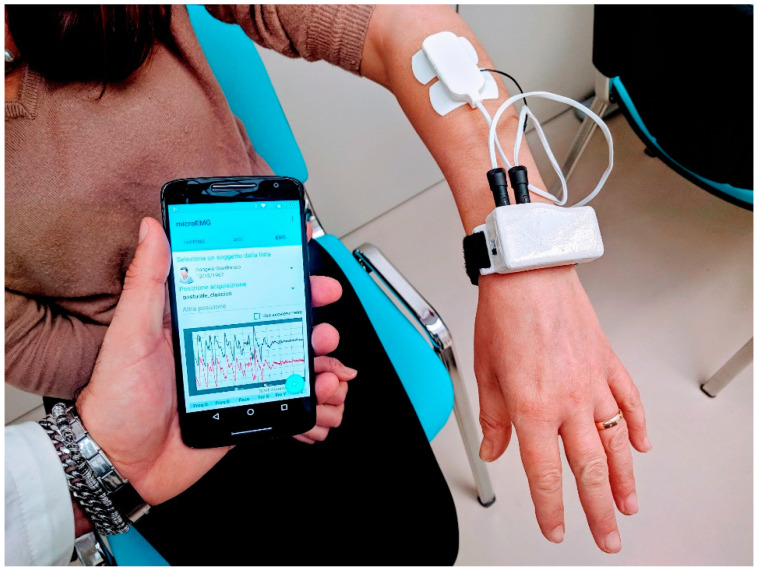
µEMG experimental system: wearable device and mobile app.

μEMG—wearable mobile tremor analysis system—is an experimental device (Figure 2), based on a cheap AVR microcontroller (Atmega 32u4 on a Bluefruit 32u4 development board by Adafruit).

It was assembled in the form of a wrist-wearable watch, with connectors for two wired muscle analog sensor boards. Each sensor board had three electrodes: two electrodes were used as differential inputs to an AD8221 instrument amplifier, and the third electrode was used as reference. The two muscle sensors, based on the Myoware development board project [21], were stuck on two antagonist muscles (extensor and flexor) of each patient’s arm. The raw signal was then rectified and integrated. Signals were sampled at 200 Hz, with 10 bit resolution, using two analog inputs of the Atmega 32u4. Samples were sent to a mobile device (smartphone) using Bluetooth Low Energy, and signal processing was performed by a custom analysis app running on an Android system. A digital Butterworth band-pass filter was applied in the 1–10 Hz band, in order to isolate the main tremor signal on each of the two channels. Tremor frequency was identified on power spectra of filtered signals. Tremor amplitude and phase were evaluated on the discrete Fourier transforms of the unfiltered signals, and the phase difference between extensor and flexor muscles was calculated, for each subject, at the tremor characteristic frequency. Signals acquired from Keypoint EMG were sampled at 6 KHz. Exported signals were analysed by replicating the same processing flow as with µEMG. The paired *t*-test was used to compare measurements of tremor frequency and phase difference acquired from the two systems, while Pearson correlation and intraclass correlation coefficient were used to assess reliability between successive measurements from µEMG. Data analysis was performed using GNU Octave and R computing environments.

## 3. Results

The comparison between the parameters measured by μEMG versus those evaluated from EMG recordings in the whole sample of Parkinson’s disease and rET patients showed no significant difference in tremor frequency, *p* = 0.57; and phase difference, *p* = 0.82. (Table 2).

**Figure 3:**
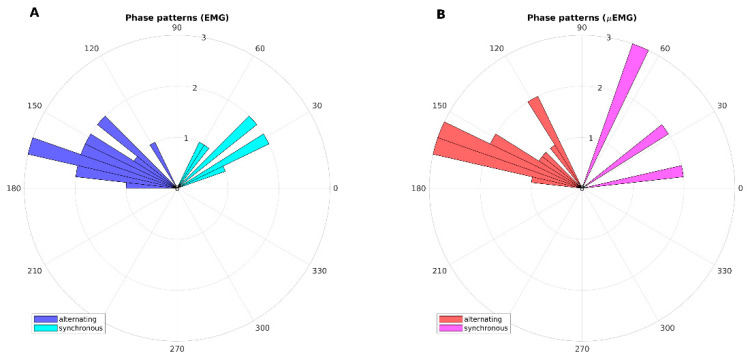
Polar histograms showing phase patterns (phase differences) evaluated with: (**A**) Dantec Keypoint EMG system; (**B**) µEMG experimental wearable device. EMG: electromyography.

In Figure 3, phase patterns are shown by means of polar histograms. The alternating and synchronous patterns detected using classical EMG were confirmed by μEMG.

**Figure 4:**
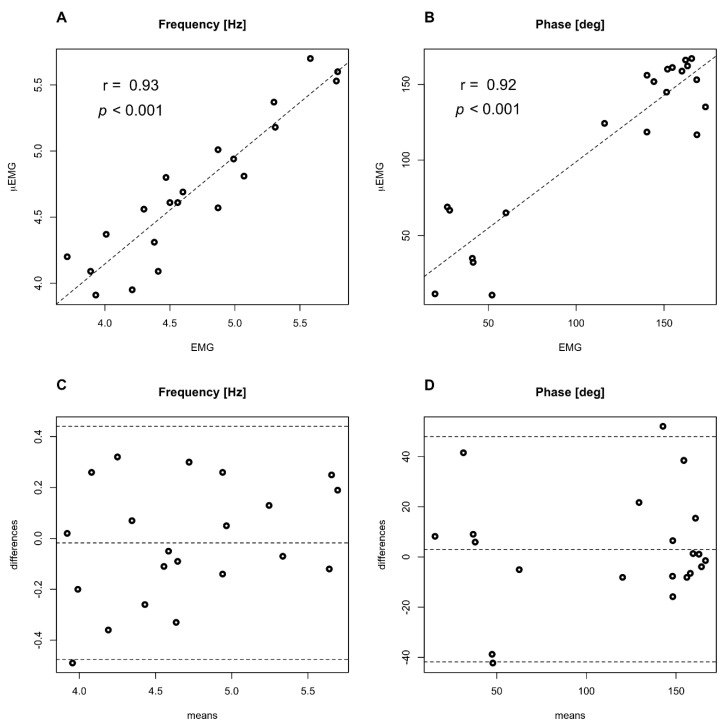
Comparison of measurements from Dantec Keypoint EMG and µEMG experimental wearable device. Correlation plots for (**A**) Frequency and (**B**) phase differences; Bland–Altman plots with 95% confidence interval for (**C**) Frequency and (**D**) phase differences. EMG: electromyography.

Correlations and Bland–Altman plots are given in Figure 4. A good degree of correlation is found for tremor frequency (A) (r = 0.93, *p* < 0.001) and phase difference (B) (r = 0.92, *p* < 0.001). Bland–Altman plots show a good level of agreement between the two techniques for tremor frequency (C) and phase difference between flexor and extensor muscles, evaluated at tremor frequency (D).

Measurements from the μEMG wearable system were taken twice on the same subjects, in order to evaluate test-retest reliability, and a very good level of agreement was found between successive measurements (Pearson correlation: r = 0.994, *p* < 0.0001; Intraclass correlation coefficient ICC = 0.992, with 95% confidence interval 0.976–0.997). The root mean square error (RMSE) between the μEMG wearable system and Dantec Keypoint EMG for phase measurements was 22.5°, while RMSE for frequency measurements was 0.23 Hz. Maximum absolute errors (MAE) for phase and frequency measurements were 52° and 0.49 Hz, respectively. We observed a relatively high MAE on phase measurements. However, this did not alter the agreement in pattern classification: all subjects with rET showed a phase difference below 90° and all subjects with tPD showed a phase difference above 90°, with both measurement systems. We also observed a higher standard deviation of phase measurements in ET subjects using μEMG, with respect to classical EMG. However, an F test showed no significant difference between standard deviations (F = 0.32, *p* = 0.19).

## 4. Discussion

In this study, we compared an innovative mobile device for the analysis of resting tremor with standard EMG recordings. We demonstrated that tremor-dominant frequency and phase pattern measured with the μEMG were superimposable to the values acquired with the standard EMG assessment.

Several methods have been proposed to assess tremor characteristics in patients with PD and ET in resting position. The gold standard for the evaluation of tremor is EMG study with surface electrodes, and the most studied parameter is the frequency. In a previous study, we demonstrated that the activation pattern of antagonistic muscles in the more affected arm with rest tremor was synchronous in patients with rET, while it was alternating in patients with tPD, thus making a distinction between patients with these diseases on an individual basis [4]. Such distinction is not possible when using phase evaluated on long-term recordings, during the occurrence of other kinds of tremor, as done in [5].

Unfortunately, standard EMG is expensive and time-consuming, needs expertise, and can be performed only by skilled specialists and technicians. Moreover, phase pattern assessment is usually performed qualitatively, by visually inspecting signals, unless traces are exported and analysed offline.

In this study, we confirmed that the phase of resting tremor in patients with tPD is alternating, while it is synchronous in patients with rET, and that the new instrument allowed us to evaluate tremor pattern parameters automatically, quickly, and with a similar performance to that obtained with EMG. To date, our device is the only wearable and mobile solution for a quick, real-time and quantitative assessment of alternating or synchronous tremor pattern. Quantitative evaluation of phase pattern allows the clinical operator to score tremors according to a grading of synchronicity (in the range of 0–90°) or non-synchronicity (alternating pattern) (in the range of 90–180°).

Our results support the validation of an innovative low-cost and easy-to-use device which allows a rapid evaluation of disease progression and quantification of tremor characteristics. Clinicians, and even general practitioners, may easily measure, by using this new device, tremor characteristic in ambulatory clinical settings, thus identifying tPD or rET patterns without the need for more expensive examinations.

## 5. Conclusions

A new simple, cheap, easy-to-use and reliable mobile device has been introduced for the evaluation of tremor frequency and phase difference at tremor frequency in subjects affected with Parkinson’s disease and in subjects with rET, in resting position. Measurements are fully automated, operator-independent, repeatable, and consistent with those acquired from classical EMG, thus allowing µEMG to be used in routine clinical practice.

### Future Work

In order to further validate the usefulness of µEMG in clinical practice, the device will be tested on a larger cohort of patients with tPD and patients with rET. Moreover, µEMG will be implemented in a new fully wireless version, with slave EMG sensor boards connected to the master bracelet through a dedicated RF communication protocol.

## Figures and Tables

**Table 1:** Demographic and clinical data of tPD patients.

	tPD (*n* = 14)	rET (*n* = 7)
Sex, M/F	9/5	4/3
Age, y	67.7 ± 9.6	67.4 ± 9.8
MMSE	27.3 ± 1.6	25.8 ± 2.1
Age at onset, y	65.1 ± 8.3	57.5 ± 8.1
Disease duration, y	3.1 ± 2.3	9.9 ± 7.8
UPDRS-ME OFF Drug	20.9 ± 9.9	7.6 ± 5.9
resting tremor subscore	4.6 ± 1.6	3.5 ± 1.4
Contralateral putamen	2.2 ± 0.3	4.1 ± 0.4
Ipsilateral putamen	2.6 ± 0.5	4.3 ± 0.5

MMSE: Mini-Mental State Examination; UPDRS-ME: Unified Parkinson’s Disease Rating Scale—Motor Examination.

**Table 2:** Comparison of frequency and phase difference measurements in patients with Parkinson’s disease and in patients with rET.

		Natus EMG	μEMG	*p* Value
PD	Frequency, [Hz]	4.63 ± 0.58	4.62 ± 0.49	0.94
	Phase difference, [°]	154.4 ± 15.3	148.3 ± 17.6	0.26
rET	Frequency, [Hz]	4.82 ± 0.71	4.88 ± 0.64	0.37
	Phase difference, [°]	38.4 ± 14.5	41.5 ± 25.6	0.79

PD: Parkinson’s disease; rET: essential tremor with resting tremor.

## Data Availability

The data presented in this study are available on request from the corresponding author. The data are not publicly available due to privacy restrictions.
